# Genome-Wide Transcriptome Profiling Reveals Genes Associated with Meiotic Drive System of *Aedes aegypti*

**DOI:** 10.3390/insects10010025

**Published:** 2019-01-10

**Authors:** Dongyoung Shin, Susanta K. Behura, David W. Severson

**Affiliations:** 1 Florida Medical Entomology Laboratory, Department of Entomology and Nematology, University of Florida, Vero Beach, FL 32962, USA; dshin@ufl.edu; 2 Department of Biological Sciences, University of Notre Dame, Notre Dame, IN 46556, USA; 3 Division of Animal Sciences & Informatics Institute, University of Missouri, Columbia, MO 65211, USA; behuras@missouri.edu

**Keywords:** Culicidae, microarray, expression profiling, sex ratio distortion

## Abstract

*Aedes aegypti* is an important mosquito vector of several arboviruses, including dengue, yellow fever, Zika, and Chikungunya, which cause significant human morbidity and mortality globally. In certain populations of this mosquito, a native meiotic drive system causes abnormal spermatogenesis that results in highly male-biased progenies from some matings. Although the basic genetics and cytogenetics of the drive mechanism were elucidated, very little is known on a transcriptome level about how the meiotic drive phenotype is expressed in individual males. To address this question, we conducted a whole-genome microarray expression study of testes from a meiotic-drive-carrying strain (T37) in comparison with testes from a non-drive-carrying strain (RED). Based on bioinformatics analyses of the microarray data, we identified 209 genes associated with the meiotic drive phenotype that were significantly differentially expressed between the two strains. *K*-means cluster analysis revealed nine clusters, in which genes upregulated in T37 testes were assigned to five clusters and genes downregulated in T37 testes were assigned to four clusters. Our data further revealed that genes related to protein translation, phosphorylation, and binding, as well as to G-protein-coupled receptor (GPCR) and peptidase activities, are differentially upregulated in testes from males with the meiotic drive genotype. Based on pathway analysis of these differentially expressed genes, it was observed that the glycosylphosphatidylinositol (GPI)-anchor biosynthesis pathway may play a role in the meiotic drive system. Overall, this investigation enhances our understanding of whole-genome gene expression associated with the meiotic drive system in *Ae. aegypti*.

## 1. Introduction

Although considerable progress was made in understanding the functional genomics driving the response to pathogen infection in the major arbovirus vector mosquito *Aedes aegypti* [1], development of effective genetic control strategies of disease-carrying mosquito populations remains a major challenge [2]. The identification of anti-pathogen effector molecules and their utility for genetic manipulation of the mosquito host is a major part of research in this direction [3]. Exploitation of endogenous selfish genetic factors for driving effector genes into populations, as well as exogenous factors including infection by the endosymbiont *Wolbachia* [4], holds potential for population replacement of *Ae. aegypti*. In *Ae. aegypti*, an endogenous meiotic drive system was identified in some *Ae. aegypti* populations, wherein an active driver distorts meiosis to give rise to highly male-biased progenies [5,6,7,8]. However, the precise molecular mechanism of how the drive system functions to cause this distortion is unknown. 

In *Ae. aegypti*, sex is determined by an autosomal locus on chromosome 1 [9]. The males are the heterogametic sex, wherein the male-determining allele (M) of the gene is dominant to the female-determining allele (m). Hickey and Craig [10] showed that the sex locus of *Ae. aegypti* is linked to the meiotic drive gene (MD). The interaction of the MD gene product with a responder locus (m) that is in linkage disequilibrium and carrying a drive sensitive allele (m^s^) results in breakage of the m^s^ allele-carrying chromosome during meiosis [11]. *Ae. aegypti* populations showing variable sex ratio distortion are known to have variations in sensitive (m^s^) and insensitive alleles (m^i^) [8,12,13]. Cytological studies of pupal testes of T-30, an *Ae. aegypti* strain that carried a meiotic drive system, revealed high incidence of chromosome breakage in the chiasmatic arm of chromosome 1 bivalents by the end of anaphase I of meiosis [11,13]. Further, evidence that the preponderance of breaks occurred in the female-determining chromosome was confirmed by Giemsa C-banding [14]. It was then suggested that the drive mechanism may be associated with crossing-over [11]. We confirmed that the meiotic drive gene is linked to the male-determining locus on chromosome 1 with ∼5.2% recombination between the two loci, and further delineated the drive gene to a ~6.5 centiMorgan interval on chromosome 1 [15]. 

The *Ae. aegypti* T37 population was selected for a strong meiotic drive gene [7]. It was observed from that study that crosses between T37 males and m^s^m^s^ females produced an average of 85% male progeny. Conversely, the RED strain of *Ae. aegypti* was shown to be highly sensitive to the meiotic drive gene [7,16]. However, the molecular basis for the MD drive system is unknown. In an earlier study, we conducted a subtractive complementary DNA (cDNA) approach to identify differentially expressed transcripts between testes of the T37 and RED strains [17]. That study identified a total of 171 unique differentially expressed transcripts that were specific to the T37 strain. Here, we performed a genome-wide whole-transcriptome analysis of testes isolated from the T37 and RED strains to identify the genes (and their functional attributes) that are differentially expressed between the two strains. In addition, we examined the distribution patterns of these genes among the three chromosomes of *Ae. aegypti*. 

## 2. Materials and Methods 

### 2.1. Mosquitoes and Tissue

The *Ae. aegypti* T37 and RED laboratory strains [7] were used in this study. Larvae for each strain were reared on a suspension of bovine liver powder and kept in an environmental chamber at 26 °C, 80% humidity, and a 16-h light/8-h dark cycle with a 60-min crepuscular period at the beginning and end of each cycle following our standard conditions [18]. 

Testes were dissected from pupae of each strain as fragmentation of chromosome 1 was shown to occur during spermatogenesis in the pupal stage [11]. Dissections were performed on glass slides kept on ice. Approximately 350 testes each from the T37 and RED strains were collected separately in 1.5-mL tubes containing RNAlater^TM^ (Invitrogen, Carlsbad, CA, USA) on ice. Tissue extraction was performed by placing pupae on a chilled glass slide, adding a drop of saline, and using an insulin needle to separate the testes from the abdominal segments. The testes samples were then homogenized with sterilized plastic pestles in TRIZOL^TM^ reagent (Invitrogen) after removing the RNAlater^TM^ reagent. Standard phenol–chloroform extractions were then performed and the RNA was precipitated with isopropanol. The precipitate was resuspended in RNAse-free water. Three biological replicates of RNA were prepared from testes of each of the two strains. 

### 2.2. Array Design and Hybridization

We used oligonucleotide microarrays designed with 60-mer oligos specific to 16,092 genes of gene build AaegL1.1 [19] as described in Behura et al. [20]. For each *Ae. aegypti* sample, hybridizations were performed with the three independent biological samples (T37-I, T37-II, T37-III, RED-I, RED-II, and RED-IIII).

### 2.3. Identification of Differentially Expressed Genes Between T37 and RED

The gene expression data corresponding to 16,092 annotated gene transcripts were normalized using the Robust Multichip Average (RMA) algorithm [21]. These include unique genes and associated alternate splice forms. Significantly differentially expressed genes (DEGs) between testes from T37 and RED strain males were determined using Student’s *t*-test with the Benjamini and Hochberg false discovery rate multiple testing correction [22]. The significance threshold (*p*-value) was 0.05 (i.e., −log_10_*p* threshold at 1.3). 

### 2.4. Cluster Analysis and Analysis of Variance of Gene Expression

The differentially expressed genes identified were further investigated to determine patterns of gene expression changes. The *k*-means cluster analysis [23] was performed to predict the expression clusters by Euclidean distance measure of expression variation with number of clusters = 10 and number of iterations = 100. DEGs within clusters were placed to individual chromosome positions using the AaegL5 genome assembly [24]. As the MD locus is on chromosome 1 [10,15], we investigated whether DEGs within individual gene clusters or in total were significantly associated with chromosome 1 versus chromosomes 2 or 3 using a hypergeometric probability test [25].

### 2.5. Functional Annotation of Differentially Expressed Transcripts

The predicted gene ontology (GO) of *Ae. aegypti* genes was obtained from VectorBase [24] using the Biomart tool. The interpro protein domains predicted from the gene sequences were also obtained using the Biomart tool. The Kyoto Encyclopedia of Genes and Genomes (KEGG) orthologs and pathways associated with *Ae. aegypti* genes were obtained from the KEGG website for *Ae. aegypti* [26]. The cumulative hypergeometric probability was used to determine statistically significant enrichment of specific GO, interpro domains, and KEGG pathways in the differentially DEGs compared to the 16,092 transcripts included in the entire microarray. All statistical analyses were performed using the R software [27]. Pathway mapping of DEGs was done using the KEGGmapper tool [26].

### 2.6. Gene Expression Analysis by Quantitative Real-Time PCR

To validate our microarray results, we performed real-time quantitative PCR (RT-qPCR) for nine randomly selected DEGs that were shown to be upregulated in T37 strain testes. All RT-qPCR assays performed in MicroAmp Optical 96-well Reaction Plates (Applied Biosystem, Foster City, CA, USA). RNAs samples from T37 strain testes were used to perform first-strand cDNA synthesis using Superscript II Reverse Transcriptase (Invitrogen) following the manufacturer’s protocol. Primer Express 3.0 software (Applied Biosystems, Foster City, CA, USA) was used to design all the primers for PCR. The RT-qPCRs were performed in a total volume of 25 µL, containing 12.5 µL of SYBR Green PCR Master Mix, 10 ng of template, 300 nmol of each primer, and nuclease-free water. Reactions were performed with the following conditions: 50 °C for 2 min, 95 °C for 10 min, followed by 40 cycles of denaturation at 95 °C for 15 s and annealing and extension at 60 °C for 1 min. The *Ae. aegypti* ribosomal gene *RpS17* [28] was used as the endogenous control gene. Our previous microarray studies confirmed no expression bias in *RpS17* across multiple treatment conditions [20,29,30]. PCR efficiency was estimated for each primer pair by determining the slopes of standard curves obtained from serial dilution analysis of the cDNA samples to ensure that the PCR efficiency was above 95% [31]. Relative expression values were obtained using the delta–delta cycle threshold (∆∆C_T_) method as described by Livak and Schmittgen [32]. The comparison of expression changes of genes between RT-qPCR assays and microarray experiments was performed by calculating Spearman’s rank correlation of fold changes of expression in T37 relative to RED strain. This analysis provides a nonparametric evaluation of rank values between two variables.

## 3. Results

### 3.1. Identification of Differentially Expressed Transcripts

**Figure 1:**
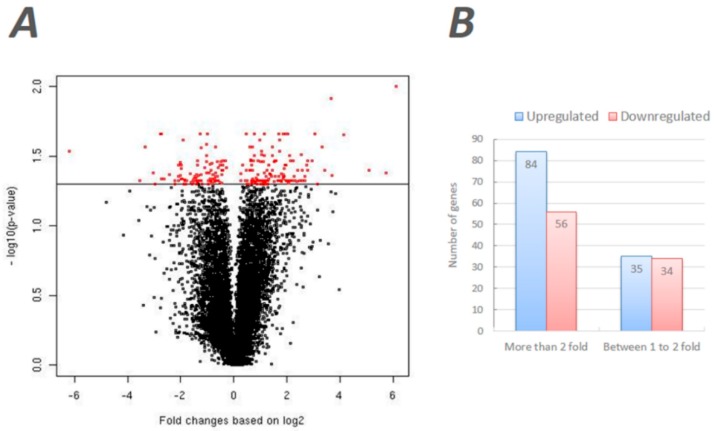
Microarray gene analysis of T37 and RED testes transcriptomes. (**A**) Volcano plot of normalized fold changes of gene expressions (*x*-axis) with the normalized *p*-values (*y*-axis) of differential expression of the corresponding genes between T37 and RED. The red dots represent the differentially expressed genes, while the black dots are not significant. The 119 genes (left side to 0) and the 90 genes (right side to 0) are positively and negatively expressed in T37 relative to RED respectively. (**B**) Relative expression levels in T37 testes in comparison to RED testes.

The expression data of these microarray experiments are available at Gene Expression Omnibus (GEO) database [33] under the accession #GSE43562. A total of 209 transcripts were identified as differentially expressed transcripts (DEGs) based on the threshold significance (*p* < 0.05) and fold change (>1.2) (Figure 1A). A complete list of significant DEGs is provided in Table S1. Of these, 119 DEGs were upregulated in the T37 testes compared to the RED strain, and the remaining 90 DEGs revealed the opposite pattern of expression. A total of 84 DEGs showed higher than twofold upregulated expression and 56 DEGs showed higher than twofold downregulated expression in T37 testes in comparison to RED testes. Nearly an equal number of DEGs showed moderate upregulation (*n* = 35) or downregulation (*n* = 34) in T37 compared to RED (Figure 1B). Furthermore, we identified 25 DEGs that were previously identified as enriched in a T37-specific cDNA library constructed using the suppressive subtraction hybridization technique [17] (Table S2). Although these DEGs showed significant differential expression in the current study, the observed expression differences between T37 and RED strain testes were marginal (<1.2 fold). Furthermore, 20 of the 25 DEGs (80%) showed significant upregulation in T37 testes as expected, while the remaining five were downregulated. Of note, two of the upregulated DEGs in this group are Ras subfamily-related (AAEL009887, wd-repeat protein) or Ras superfamily-related (AAEL009884, Ran-binding protein) genes. Still, we elected to maintain a more stringent fold change threshold (>1.2) and, thus, did not include them in subsequent analyses for the current study. 

**Figure 2:**
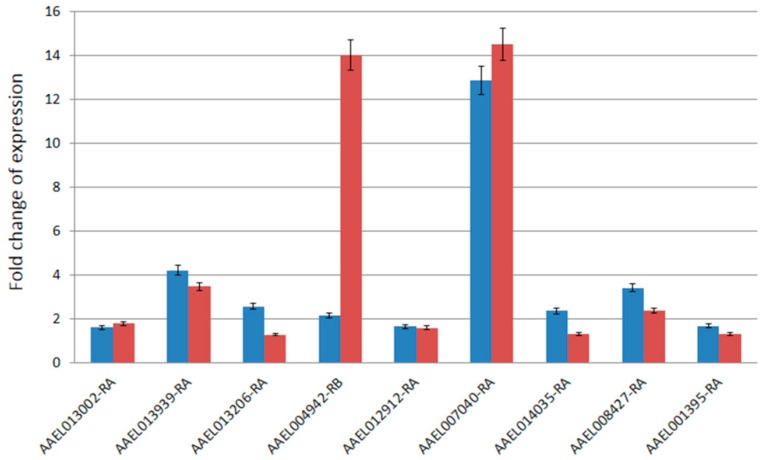
Comparison of fold-changes of expression of nine genes between microarray data (blue) and RT-qPCR data (red). The x-axis shows the VectorBase ID of the nine genes and y-axis shows the fold changes of expression. The error bars shown represent standard error of data.

The RT-qPCR assays were performed to validate the expression patterns observed from the microarray experiments. A total of nine genes were randomly selected for RT-qPCR from the significantly upregulated genes in T37 testes identified from the microarray data. The fold changes in expression of the nine genes observed from the RT-qPCR assays were compared with that of the microarray data (Figure 2). In general, the results were consistent between the RT-qPCR and the microarray data (Spearman’s rank correlation = 0.65). One of the transcripts (AAEL004942-RB) showed much higher (14-fold) expression in the RT-qPCR data compared to only 2.1-fold difference in the microarray data. The other eight genes showed highly similar (Spearman’s rank correlation = 0.99) expression changes in both microarray and RT-qPCR experiments. 

### 3.2. Correlated Expression Patterns of DEGs

Based on the Euclidean distance measure of expression changes among the DEGs, nine expression clusters were identified by the *k*-means clustering method, wherein the genes within each cluster revealed similar expression pattern among the six biological samples (Table 1). The patterns of expression variation of genes within clusters are shown in Figure S1. A complete list of gene identifiers (IDs) and cluster associations is provided in Table S1. Apart from these nine clusters, another cluster was also identified but excluded from further analysis due to low sample size (five genes total).

We were able to place a total of 198 DEGs to unique chromosome positions (Table S1) using the recent AaegL5 genome assembly [24]. As the meiotic drive gene was mapped to chromosome 1 [6,15], we wanted to investigate whether the DEGs within individual clusters or in total showed any bias in chromosome distribution. Based on the cumulative hypergeometric probabilities, we observed that DEGs were not significantly overrepresented in chromosome 1 or any of the other chromosomes (*p* > 0.05). 

### 3.3. Functional Characteristics of DEGs

To characterize the functions associated with the DEGs, a gene ontology (GO) analysis was performed. Based on the GO term annotation of *Ae. aegypti* genes, the DEGs were found to be associated with 178 unique GO terms. For each GO term represented by DEGs, the numbers of gene associations were compared with all predicted genes of *Ae. aegypti* to determine if specific terms were enriched among DEGs (Table 2). Each of these GO terms revealed significant (*p* < 0.05, hypergeometric test) enrichment in DEGs compared to the all the genes that represented the microarray. Of these, only three functions relating to extracellular region, hydrolase activity, and proteolysis showed significant (*p* < 0.05) overrepresentation among the genes that were upregulated in the T37 testes. The functional annotations of genes represented by the individual DEGs were also predicted from the enrichment of specific GO terms in each cluster (Table 3). It was found from this analysis that the genes within individual clusters perform similar functions. For example, cluster C genes are mostly associated with protein phosphorylation activities, whereas genes of cluster D are predominantly involved in peptidase and proteolysis functions. The G-protein-coupled receptor (GPCR) genes are mostly associated with cluster I.

We also mapped the DEGs to KEGG [26] pathways of *Ae. aegypti* and found that the DEGs mapped to 24 different pathways. Based on rank order of numbers of genes mapped to each pathway, the metabolic pathway (KEGG ID aag01100) was the highest ranking among all the 24 pathways. A total of nine DEGs between T37 and RED were related to this KEGG pathway (Table 4). Of these, five genes mapped specifically to the glycosylphosphatidylinositol (GPI)-anchor biosynthesis (KEGG ID aag00563) metabolic pathway, as they belonged to the unique KEGG orthology group K05286.

## 4. Discussion

In this investigation, we performed microarray experiments to compare genome-wide transcriptional changes in testes of the *Ae. aegypti* T37 strain that was previously selected for a strong meiotic driver in comparison to testes of the RED strain that is sensitive to meiotic drive. We examined testes at the early pupal stage of both T37 and RED strains because chromosome breakage was shown to occur between the fourth larval stage and adult stages [11]. Targeting gene expression changes in testes was reasonable based on the fact that the meiotic drive gene is associated with the male-determining chromosome 1 [6,15]. The typical sex ratio in *Ae. aegypti* progeny from individual females is approximately 1:1; however, progeny from females that mate with males carrying the endogenous meiotic drive gene coupled with the male-determining allele at the sex locus, and a drive-sensitive allele at the responder locus coupled with the female-determining allele show severe sex ratio distortion with very few females in their offspring [7,10]. This effective driver in *Ae. aegypti* causes sensitive responder-bearing chromosome breakage during spermiogenesis [11,34]. 

The *Ae. aegypti* meiotic drive system shows similar meiotic drive systems to *Drosophila melanogaster* and mouse because they are similarly activated in testes during spermatogenesis and are controlled by a distorter gene and a responder locus [35,36]. The *D. melanogaster* and mouse meiotic drive genes are best characterized for the segregation disorder (SD) and t-complex, respectively. It was shown that a truncated duplicated *RanGAP* gene is the effector gene for SD in *D. melanogaster* [35]. It is also known that the defective RanGAP leads to chromatin condensation failure during spermatid maturation [37,38]. In *Ae. aegypti*, the *RanGAP* gene is upregulated in T37 testes but is unlikely to be the meiotic drive gene based on mapping results [39]. The mouse t-complex causes abnormal flagella function of sperm and distorts chromosome 17 transmission in the progenies. The effector gene of the t-complex encodes guanosine triphosphate hydrolase (GTPase)-activating protein (GAP), called T-cell-activating Rho GTPase-activating protein (*Tagap1*) and is highly similar to *Rho* GTPase-activating protein tandem repeat [40]. The two meiotic drive systems, t- and SD complex are GAP proteins in the Ras superfamily signaling pathway. Thus, there is potential that the Ras genes differentially expressed between T37 and RED may also have a role in expression of the meiotic drive system in *Ae. aegypti* populations.

Understanding the genetic components associated with expression of the meiotic drive phenotype in *Ae. aegypti* has implications for the development of transgenic approaches to prevent pathogen transmission. Cage trials suggested that release of *Ae. aegypti* males carrying a strong MD gene into drive-sensitive populations has potential as an effective tool for population replacement [41]. However, computer simulations studies showed that the stability of the drive system may be affected by several intrinsic, as well as extrinsic, factors [42]. At the same time, rapid selection for tolerance (i.e., drive suppressor) genes was reported for the *Ae. aegypti* drive system in some studies [16]. However, no evidence of similar selection for tolerance was observed in long-term cage trials [41]. Thus, our primary assumption of the current study was that the expression of meiotic-drive phenotype in *Ae. aegypti* might be more complex than presently thought. Hence, a genome-scale dissection of gene expression patterns in sensitive versus insensitive strains to the drive system was necessary. We hypothesized that the differentially expressed genes from genome-wide investigation may reveal the genes that were limited from our earlier investigation using the cDNA analysis method [17].

We predicted function of DEGs by analyzing GO terms and KEGG pathways associated with the genes. The glycosylphosphatidylinositol (GPI)-anchor metabolic process was one of the overrepresented functions associated with the DEGs. The GPI anchor is a glycolipid that has a key function in posttranslational modification of proteins. After the attachment to the C-terminal of proteins, the GPI-anchored proteins are activated to interact with the Ras signaling pathway and calcium transport [43]. Our array data showed that genes related to the phosphoinositide 3-kinase (PI3K) cascade, endocytic process, and immune response, involving endosomal growth factor receptor (EGFR) were upregulated in the T37 strain compared to the RED strain (Table S3). It is known that the Ras GTPases regulate PI3K activities [44,45]. The role of Rap and Ras GTPase in regulating cell proliferation, differentiation, and adhesion mechanisms was demonstrated [46]. In mosquitoes, they are known to be involved in regulation of innate immunity [47,48], as well as in gonadotrophic cycles [49,50]. Hence, it is likely that the GPI-anchor metabolic process overrepresented in DEGs from our array data may play a key role in interacting with the Ras signaling pathway in *Ae. aegypti* in expressing the meiotic drive phenotype in *Ae. aegypti*.

## 5. Conclusions

Whole-transcriptome comparisons of testes dissected from *Ae. aegypti* males from a meiotic-drive-carrying strain (T37) in comparison with testes from a non-drive-carrying strain (RED) identified 209 DEGs between the two strains. Nine clusters of DEGs were identified, wherein the genes within each cluster revealed similar expression patterns. Five and four of these clusters showed significant upregulation in T37 testes and RED testes, respectively. Genes related to the phosphoinositide 3-kinase (PI3K) cascade, endocytic process, and immune response involving endosomal growth factor receptor (EGFR) were upregulated in the T37 strain compared to the RED strain. No association of DEGs with individual chromosomes was observed.

## Figures and Tables

**Table 1:** Number of differentially expressed genes (DEGs) and the expression pattern within individual clusters.

Cluster Name	Number of DEGs	Expression Pattern
CLUSTER-A	20	Upregulated in T37
CLUSTER-B	29	Upregulated in T37
CLUSTER-C	23	Upregulated in T37
CLUSTER-D	20	Downregulated in T37
CLUSTER-E	17	Upregulated in T37
CLUSTER-F	23	Downregulated in T37
CLUSTER-G	14	Downregulated in T37
CLUSTER-H	13	Upregulated in T37
CLUSTER-I	45	Downregulated in T37

**Table 2:** List of gene ontology (GO) terms with significant (*p* < 0.05) enrichment among the differentially expressed genes compared to all annotated genes in the genome. The terms underlined are enriched among the upregulated genes in T37 testes compared to the all the DEGs.

GO Term	Number of Genes
ATP binding	7
catalytic activity	10
extracellular region	8
hydrolase activity	7
integral to membrane	15
membrane	12
metabolic process	12
metal-ion binding	17
nucleic-acid binding	9
nucleotide binding	7
nucleus	11
oxidation–reduction process	10
oxidoreductase activity	9
proteolysis	7
ribosome	6
structural constituent of ribosome	6
translation	6
transport	7
zinc-ion binding	17

**Table 3:** Functional annotation of genes of predicted clusters. The number of DEGs associated with the GO in comparison to the total cluster genes of the same GO term is shown.

CLUSTER	GO Term	Number of Genes
CLUSTER-A	ribosome	4 out of 6
	structural constituent of ribosome	4 out of 6
	translation	4 out of 6
CLUSTER-B	catalytic activity	5 out of 10
	protein binding	1 out of 24
CLUSTER-C	protein kinase activity	2 out of 2
	protein phosphorylation	2 out of 2
	protein serine/threonine kinase activity	2 out of 2
	transferase activity, transferring phosphorus-containing groups	3 out of 4
CLUSTER-D	carboxypeptidase activity	2 out of 2
	metallopeptidase activity	2 out of 2
	peptidase activity	2 out of 2
	proteolysis	4 out of 7
CLUSTER-F	binding	3 out of 4
CLUSTER-I	G-protein-coupled receptor activity	3 out of 3
	G-protein-coupled receptor signaling pathway	3 out of 3
	transferase activity, transferring acyl groups other than amino-acyl groups	3 out of 3

**Table 4:** List of DEGs related to Kyoto Encyclopedia of Genes and Genomes (KEGG) metabolic pathways. The expression pattern of these genes in T37 testes and their association with predicted expression clusters are shown. The genes with asterisks map to the specific metabolic pathway of glycosylphosphatidylinositol (GPI)-anchor biosynthesis (KEGG identifier (ID) aag00563).

Gene	Expression in T37	Cluster #
AAEL004575	Upregulated	Cluster-A
AAEL002465*	Upregulated	Cluster-C
AAEL007159	Upregulated	Cluster-C
AAEL011190*	Upregulated	Cluster-C
AAEL015107*	Upregulated	Cluster-C
AAEL002331	Downregulated	Cluster-D
AAEL007498*	Downregulated	Cluster-F
AAEL008875*	Downregulated	Cluster-F
AAEL009745	Downregulated	Cluster-G

## Supplementary Materials

The following are available online at http://www.mdpi.com/2075-4450/10/1/25/s1: Figure S1: Correlated expression changes of genes among the samples of T37 and RED; Table S1: List of DEGs, potential functions, and chromosome locations; Table S2: List of the 25 transcripts that show differential expression levels between T37 and RED strains from the microarray data and that were also identified as differentially expressed from a previous subtractive cDNA hybridization study; Table S3: List of differentially expressed genes identified from the array that map to phosphoinositide 3-kinase (PI3K) cascade, endocytic process and immune response involving endosomal growth factor receptor (EGFR) KEGG pathways.
