# Supplementary material for: Genome-Wide Transcriptome Profiling Reveals Genes Associated with Meiotic Drive System of Aedes aegypti
Source: Insects. 2019 Jan 10;10(1):25. doi: 10.3390/insects10010025 (PMC6358845; doi:10.3390/insects10010025)
Supplement: Supplementary file 1 [file insects-10-00025-s001.zip › insects-406071-supplementary/Table S2.docx]

**Table S2.** List of the 25 transcripts that show differential expression levels between T37 and Red strains from the microarray data and that were also identified as differentially expressed from a previous subtractive cDNA hybridization study [Shin et al. 2011].

| Gene  Transcript | Gene  Description | T37  Expr | RED  Expr | RED/T37  Expr | *p*-Value (t-test) |
| --- | --- | --- | --- | --- | --- |
| AAEL000718_RA | virus-induced RNA, putative | 16994.3 | 20485.7 | 1.21 | 0.04 |
| AAEL000840_RA | inositol 5-phosphatase | 6709.3 | 6306.0 | 0.94 | 0.02 |
| AAEL000895_RA | peroxisome biogenesis factor 1 (peroxin-1) | 20604.7 | 15082.0 | 0.73 | 0.04 |
| AAEL001537_RA | bax inhibitor | 15036.0 | 18175.3 | 1.21 | 0.04 |
| AAEL002851_RA | tubulin beta chain | 45084.0 | 50744.0 | 1.13 | 0.03 |
| AAEL003671_RA | hypothetical protein | 17882.0 | 13148.7 | 0.74 | 0.03 |
| AAEL004405_RA | conserved hypothetical protein | 6561.7 | 3795.7 | 0.58 | 0.04 |
| AAEL004984_RA | cullin-associated NEDD8-dissociated protein1 | 20045.3 | 15678.0 | 0.78 | 0.03 |
| AAEL005367_RA | myotubularin-related protein | 12064.0 | 7098.0 | 0.59 | 0.01 |
| AAEL005885_RA | arginyltransferase, putative | 9028.7 | 5658.7 | 0.63 | 0.00 |
| AAEL005885_RB | arginyltransferase, putative | 7656.0 | 4533.0 | 0.59 | 0.01 |
| AAEL005885_RC | arginyltransferase, putative | 9881.3 | 5880.0 | 0.60 | 0.01 |
| AAEL006625_RA | conserved hypothetical protein | 6390.0 | 4180.3 | 0.65 | 0.01 |
| AAEL007022_RA | tfiia large subunit | 44306.3 | 42896.0 | 0.97 | 0.04 |
| AAEL007180_RA | PFTAIRE-interacting factor 1A, putative | 7196.7 | 11912.7 | 1.66 | 0.01 |
| AAEL008862_RA | conserved hypothetical protein | 22568.3 | 19502.7 | 0.86 | 0.00 |
| AAEL009884_RA | ran-binding protein | 24580.3 | 17909.7 | 0.73 | 0.05 |
| AAEL009887_RA | wd-repeat protein | 25500.7 | 16501.3 | 0.65 | 0.02 |
| AAEL010217_RB | mediator complex, subunit, putative | 5855.7 | 3914.7 | 0.67 | 0.03 |
| AAEL010401_RA | conserved hypothetical protein | 1866.0 | 1033.3 | 0.55 | 0.02 |
| AAEL011315_RA | conserved hypothetical protein | 1314.3 | 1071.0 | 0.81 | 0.02 |
| AAEL011380_RA | high mobility group B1, putative | 10020.3 | 8069.0 | 0.81 | 0.04 |
| AAEL011406_RA | cytochrome c oxidase polypeptide | 14214.0 | 7880.0 | 0.55 | 0.02 |
| AAEL012065_RA | cpeb | 17879.3 | 11523.0 | 0.64 | 0.01 |
| AAEL014017_RA | testis-specific serine/threonine kinase 22c | 17088.7 | 19909.3 | 1.17 | 0.03 |
