# Supplementary material for: Genome-Wide Transcriptome Profiling Reveals Genes Associated with Meiotic Drive System of Aedes aegypti
Source: Insects. 2019 Jan 10;10(1):25. doi: 10.3390/insects10010025 (PMC6358845; doi:10.3390/insects10010025)
Supplement: Supplementary file 1 [file insects-10-00025-s001.zip › insects-406071-supplementary/TableS3.docx]

**Table S3.** List of differentially expressed genes identified from the array that map to phosphoinositide 3-kinase (PI3K) cascade, endocytic process and immune response involving endosomal growth factor receptor (EGFR) KEGG pathways.

| VectorBase ID | KEGG ID | Functional Description |
| --- | --- | --- |
| AAEL001133 | K17889 | Regulation of autophagy |
| AAEL002465 | K05288 | Glycosylphosphatidylinositol(GPI)-anchor biosynthesis |
| AAEL002846 | K00328 | Inositol phosphate metabolism |
| AAEL004575 | K12309 | Galactose metabolism |
| AAEL007159 | K00902 | N-Glycan biosynthesis |
| AAEL007203 | K12870 | Spliceosome |
| AAEL008828 | K01672 | Nitrogen metabolism |
| AAEL009308 | K14554 | Ribosome biogenesis in eukaryotes |
| AAEL010946 | K10723 | Insect hormone biosynthesis |
| AAEL011190 | K05287 | Glycosylphosphatidylinositol(GPI)-anchor biosynthesis |
| AAEL012989 | K14304 | RNA transport |
| AAEL013939 | K04371 | MAPK signaling pathway-fly |
| AAEL015107 | K05287 | Glycosylphosphatidylinositol(GPI)-anchor biosynthesis |
