# Supplementary figures and images for: Genome-Wide Transcriptome Profiling Reveals Genes Associated with Meiotic Drive System of Aedes aegypti
Source: Insects. 2019 Jan 10;10(1):25. doi: 10.3390/insects10010025 (PMC6358845; doi:10.3390/insects10010025)

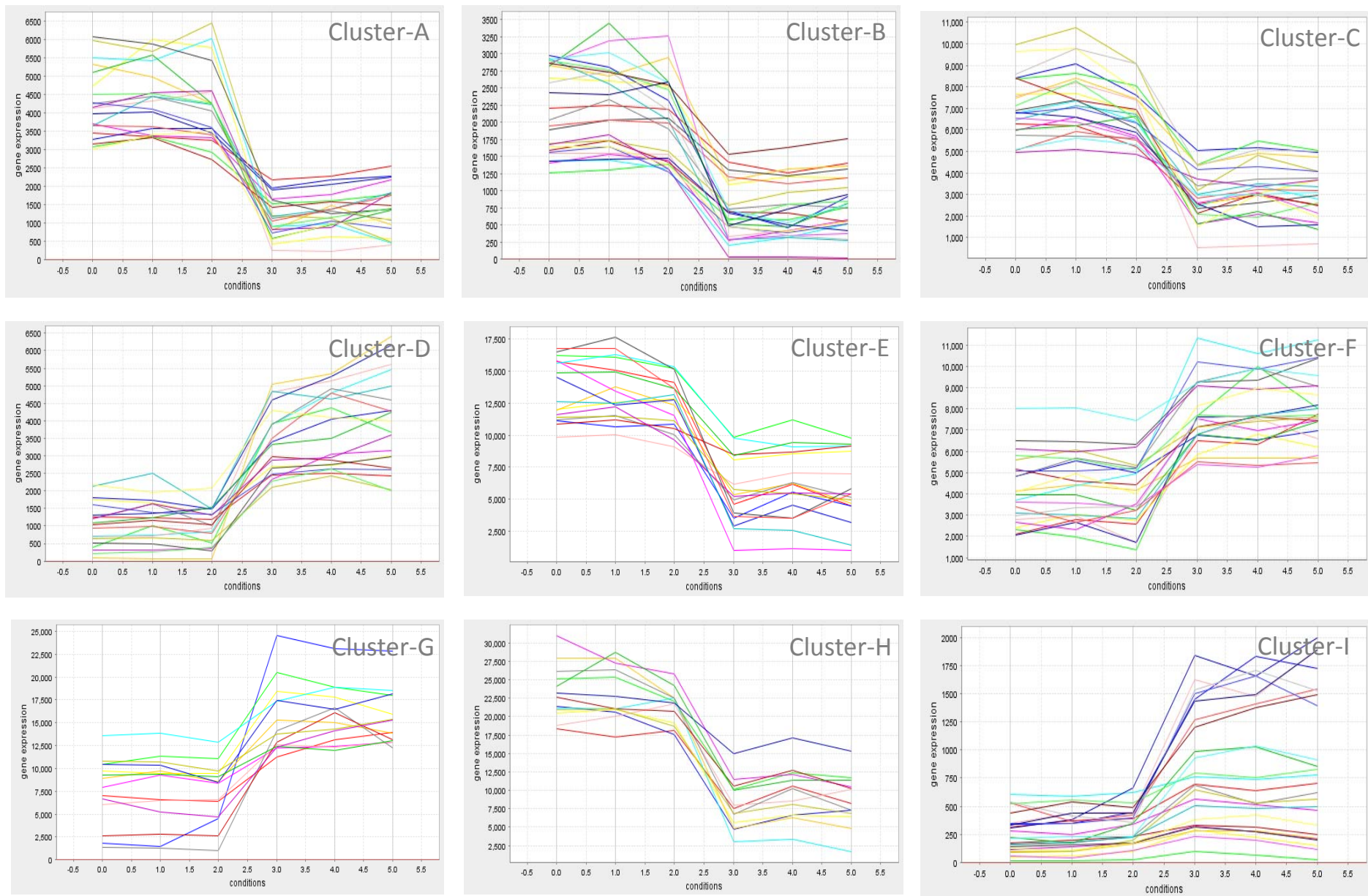

Figure S1. Correlated expression changes of genes among the samples of T37 and RED.

Supplement: Supplementary file 1 [file insects-10-00025-s001.zip › insects-406071-supplementary/Figure S1.pdf]
